# Experimental and Microstructural Assessment of High-Plasticity Clay Stabilized with Recycled PVC Powder and Polyester Fiber

**DOI:** 10.3390/polym18182274

**Published:** 2026-09-17

**Authors:** Melike Yiğit, Ahmet Erdağ, Pınar Sezin Öztürk Kardoğan

**Affiliations:** 1 Graduate School of Natural and Applied Sciences, Gazi University, 06560 Ankara, Türkiye; 2 Department of Civil Engineering, Faculty of Engineering and Architecture, Kafkas University, 36000 Kars, Türkiye; ahmet.erdag@kafkas.edu.tr; 3 Department of Civil Engineering, Faculty of Technology, Gazi University, 06560 Ankara, Türkiye; sezinozturk@gazi.edu.tr

**Keywords:** high-plasticity clay, soil stabilization, recycled PVC powder, recycled polyester fiber, unconfined compressive strength

## Abstract

High-plasticity clays present significant challenges in transportation infrastructure because of their low strength, high compressibility, and pronounced moisture sensitivity. This study investigates the effects of recycled polyvinyl chloride (PVC) powder and recycled polyester fibers, used individually and in combination, on the compaction behavior, unconfined compressive strength (UCS), and microstructural characteristics of a high-plasticity clay. PVC powder was incorporated at 5%, 10%, and 15%, while recycled polyester fibers were added at 0.5%, 0.75%, and 1.0% by dry weight of soil. Standard Proctor compaction, UCS, and scanning electron microscopy (SEM) analyses were performed to evaluate the engineering performance of the modified mixtures. The results showed that the incorporation of both PVC powder and recycled polyester fibers reduced the maximum dry unit weight of the clay, with 5% PVC and 0.5% fiber exhibiting the most favorable compaction characteristics. The specimen containing 15% PVC powder achieved the highest UCS (204.08 kPa), corresponding to an increase of approximately 108.6% compared with the untreated clay, whereas the specimen reinforced with 1.0% recycled polyester fiber exhibited an approximately 89.7% increase in compressive strength while maintaining greater deformation capacity. The hybrid mixture containing 5% PVC powder and 0.5% recycled polyester fiber produced a 53.7% improvement in UCS and exhibited a balanced stress–strain response. SEM observations revealed that PVC powder primarily enhanced the soil through physical void filling and particle rearrangement, whereas recycled polyester fibers improved the continuity of the soil skeleton through fiber bridging and mechanical interlocking. Overall, the results demonstrate that recycled PVC powder and recycled polyester fibers represent promising and environmentally sustainable stabilization materials for improving the engineering performance of high-plasticity clays used in transportation infrastructure.

## 1. Introduction

High-plasticity clays are among the most problematic geomaterials encountered in geotechnical engineering owing to their high compressibility, low bearing capacity, pronounced shrinkage–swelling potential, and strong sensitivity to moisture fluctuations. These characteristics frequently result in excessive settlements, volume instability, and premature deterioration of foundations, embankments, and pavement structures, thereby increasing maintenance costs and reducing service life. Consequently, improving the engineering behavior of high-plasticity clays has remained a long-standing research priority. In recent years, this objective has evolved beyond achieving superior mechanical performance to encompass environmentally responsible stabilization strategies that reduce the consumption of natural resources and promote the beneficial utilization of industrial and municipal wastes [1,2]. Recent studies have demonstrated that recycled polymeric fibers not only improve the mechanical behavior of clay soils but also reduce shrinkage cracking and enhance the long-term sustainability of geotechnical structures [3].

Conventional stabilizers such as cement and lime have been extensively employed because of their proven ability to improve the strength and stiffness of weak soils. However, their production is associated with considerable energy consumption and greenhouse gas emissions, stimulating increasing interest in sustainable alternatives derived from recycled materials. Among these alternatives, waste plastics have attracted particular attention because they simultaneously improve engineering performance and provide an environmentally beneficial route for recycling persistent polymeric wastes [4,5,6]. Existing evidence indicates that the effectiveness of plastic-based stabilization depends not only on polymer type but also on additive morphology, particle size, and dosage. While appropriate contents generally enhance strength, stiffness, and deformation resistance, excessive additions often reduce stabilization efficiency owing to weakened soil–particle interaction and increased structural discontinuities. These findings collectively demonstrate that the engineering performance of recycled polymers is governed primarily by the mechanisms through which they interact with the soil skeleton rather than by their chemical composition alone.

Polyvinyl chloride (PVC) represents one of the most promising recycled polymers for sustainable ground improvement because of its widespread industrial availability, durability, and high recycling potential. Previous studies have shown that recycled PVC can significantly improve compaction characteristics, swelling behavior, and California Bearing Ratio (CBR) when incorporated into clay soils at appropriate contents [7,8]. Nevertheless, nearly all existing investigations have evaluated PVC in the form of strips, sheets, or packaging fragments, whereas finely ground PVC powder has received remarkably limited attention. Unlike larger polymer inclusions, PVC powder is expected to interact directly with the soil matrix by modifying particle packing, contact conditions, and pore distribution. Similarly, recent experimental studies have shown that recycled polymer-based stabilizers improve particle packing and increase soil strength while supporting sustainable ground improvement practices [9]. Such a fundamentally different stabilization mechanism suggests that its engineering behavior cannot simply be extrapolated from studies conducted using larger recycled plastic inclusions.

Randomly distributed fiber reinforcement constitutes another well-established approach for improving problematic soils. Unlike particulate additives that primarily modify soil fabric, discrete fibers mobilize tensile resistance through frictional interaction and mechanical interlocking with surrounding particles, thereby restricting particle movement, delaying crack initiation, and improving deformation resistance [1]. Among synthetic fibers, recycled polyester has received considerable attention because it combines effective mechanical reinforcement with the sustainable reuse of plastic and textile wastes. Previous investigations consistently reported improvements in shear strength, CBR, and deformation behavior following polyester fiber inclusion, although the magnitude of improvement depends strongly on soil characteristics, fiber geometry, and reinforcement content [10,11,12,13,14]. Collectively, these studies demonstrate that recycled polyester fibers primarily improve soil performance through tensile reinforcement rather than by altering the intrinsic structure of the soil matrix. From a practical perspective, both PVC waste and recycled polyester fibers originate from widely generated plastic and textile waste streams, supporting their potential availability for geotechnical applications. Their use may also reduce dependence on virgin stabilization materials; however, the overall cost is influenced by waste collection, sorting, grinding or fiber processing, and transportation. Therefore, a detailed economic assessment is required before their large-scale application.

Recent advances in sustainable ground improvement have increasingly shifted from single-additive stabilization toward hybrid systems capable of mobilizing complementary reinforcing mechanisms within the same soil matrix. In such systems, particulate additives primarily improve the internal soil fabric by modifying particle arrangement and reducing void space, whereas fibers enhance tensile resistance, redistribute stresses, and inhibit crack propagation. Consequently, hybrid stabilization has demonstrated greater potential than individual additives for simultaneously improving strength, stiffness, and deformation characteristics. This concept has been confirmed by studies combining polyester fibers with biopolymers, cementitious binders, and nano-sized additives, where the superior performance was consistently attributed to the complementary functionality between matrix modification and mechanical reinforcement rather than to the individual contribution of each stabilizer [15,16,17,18,19]. Furthermore, the composite stabilization combining waterborne polymer bonding with recycled polypropylene fibers substantially improves unconfined compressive strength, shear resistance, and structural integrity through particle bridging and matrix reinforcement [20].

To provide a critical comparison of the existing literature, previous studies on polymer-based soil stabilization are summarized in Table 1 with particular emphasis on additive morphology, soil type, reported engineering effects, and the principal limitations relevant to the present study. The comparison demonstrates that the mechanical contribution of recycled polymers depends strongly on their physical form and reinforcement mechanism. In particular, particulate polymers and discrete fibers interact with the soil matrix through fundamentally different mechanisms, while systematic investigations combining finely divided PVC with recycled polyester fibers remain notably limited.

Despite these advances, significant knowledge gaps remain regarding the hybrid stabilization of high-plasticity clays using recycled polymeric materials. Existing investigations have predominantly focused on recycled plastics in the form of strips, flakes, granules, or packaging fragments, whereas the geotechnical potential of finely ground PVC powder remains largely unexplored. Likewise, recycled polyester fibers have been examined primarily as standalone reinforcements or in combination with conventional binders such as cement and biopolymers. Consequently, the coupled use of recycled PVC powder and recycled polyester fibers has not yet been systematically investigated. This particular combination was selected because the two recycled materials are expected to provide complementary physical mechanisms: finely ground PVC powder can modify particle packing and interparticle contacts, whereas discrete polyester fibers can provide tensile restraint, mechanical interlocking, and crack bridging. Thus, their combined use was investigated to determine whether matrix modification and fiber reinforcement could be mobilized simultaneously within high-plasticity clay. More importantly, the complementary interaction between soil-fabric modification induced by particulate recycled polymers and tensile reinforcement mobilized by discrete fibers has not been clearly elucidated. A mechanistic understanding of this interaction is essential for developing efficient and environmentally sustainable hybrid stabilization systems capable of improving both strength and deformation characteristics of problematic clay soils.

Furthermore, recent microstructural and mineralogical evaluations on fine-grained and clayey soils have highlighted that spatial particle arrangement, interparticle contacts, and mineral composition strongly control the cyclic softening, pore pressure generation, and deformation behavior under complex loading conditions [27].

Accordingly, this study experimentally investigates the individual and combined effects of recycled PVC powder and recycled polyester fibers on the engineering behavior of a high-plasticity clay. Particular emphasis is placed on elucidating the complementary mechanisms through which PVC powder modifies soil fabric and interparticle contact conditions, while recycled polyester fibers enhance tensile load transfer through mechanical interlocking and crack-bridging. The proposed hybrid stabilization system is evaluated in terms of compaction characteristics, consistency limits, strength, and deformation behavior to establish the relationship between material interaction and engineering performance. Beyond assessing the effectiveness of recycled polymeric additives, this study provides mechanistic insight into hybrid polymer–soil interaction and contributes to the development of sustainable stabilization strategies for problematic clay soils based entirely on recycled materials.

## 2. Materials and Methods

### 2.1. Materials

**Figure 1:**
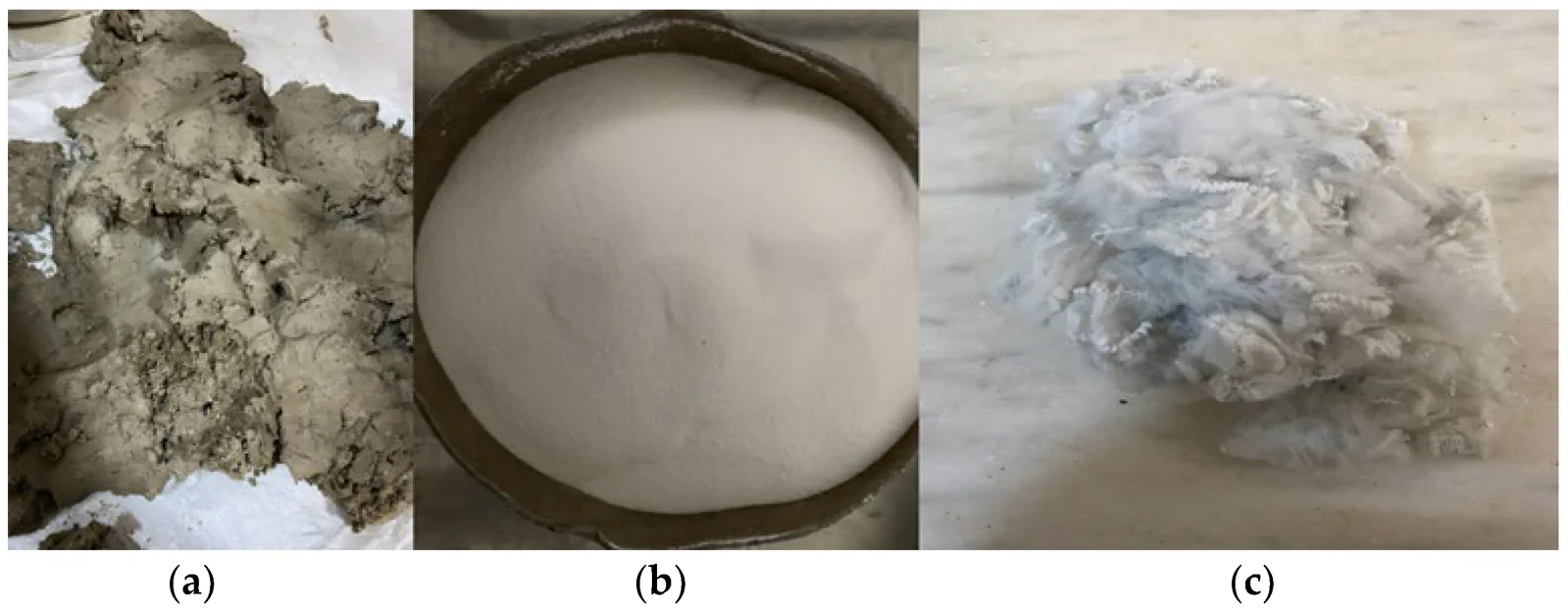
Materials used in this study: (**a**) high-plasticity clay soil, (**b**) PVC powder, and (**c**) recycled polyester fiber.

The materials used in this study consisted of a natural high-plasticity clay, PVC powder, and recycled polyester fibers. Figure 1 presents the materials used throughout the experimental program, while their physical and engineering characteristics are described in the following sections.

#### 2.1.1. Clay Soil

**Figure 2:**
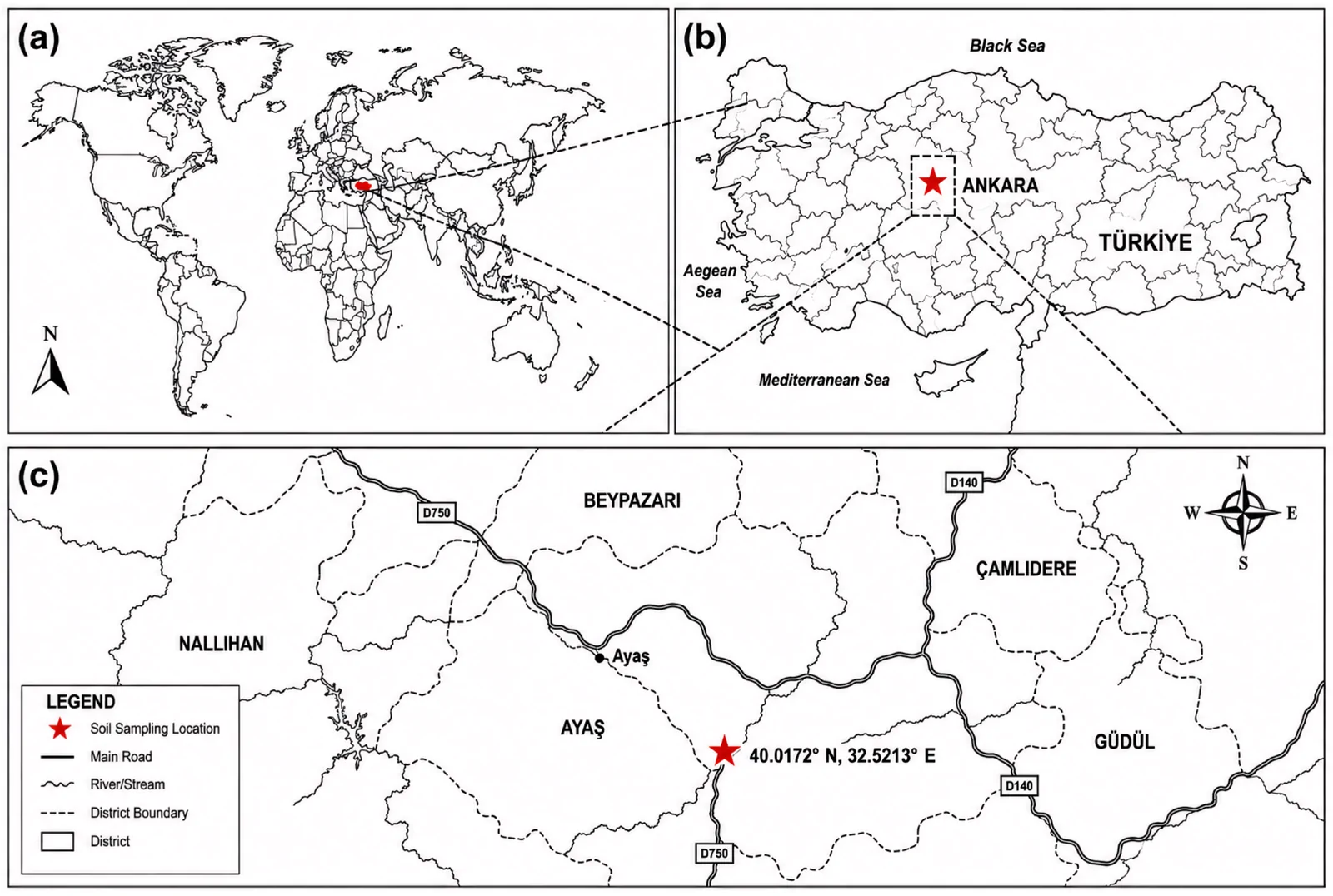
Location of the soil sampling site in (**a**) Turkey (**b**) Ankara (**c**) Yenikent Neighborhood, Sincan District [28].

The clay soil used in this study was obtained from a natural deposit located at Parcel No. 7, Block 669, Yenikent Neighborhood, Sincan District, Ankara, Türkiye (40.0172° N, 32.5213° E), as illustrated in Figure 2. The site was selected because it contains naturally occurring highly plastic clay deposits that are representative of problematic fine-grained subgrade soils commonly encountered in the region. Bulk soil samples were collected from the near-surface layer and transported to the laboratory. Prior to testing, the material was air-dried, pulverized, and passed through the required sieve to remove oversized particles and ensure uniform specimen preparation.

**Figure 3:**
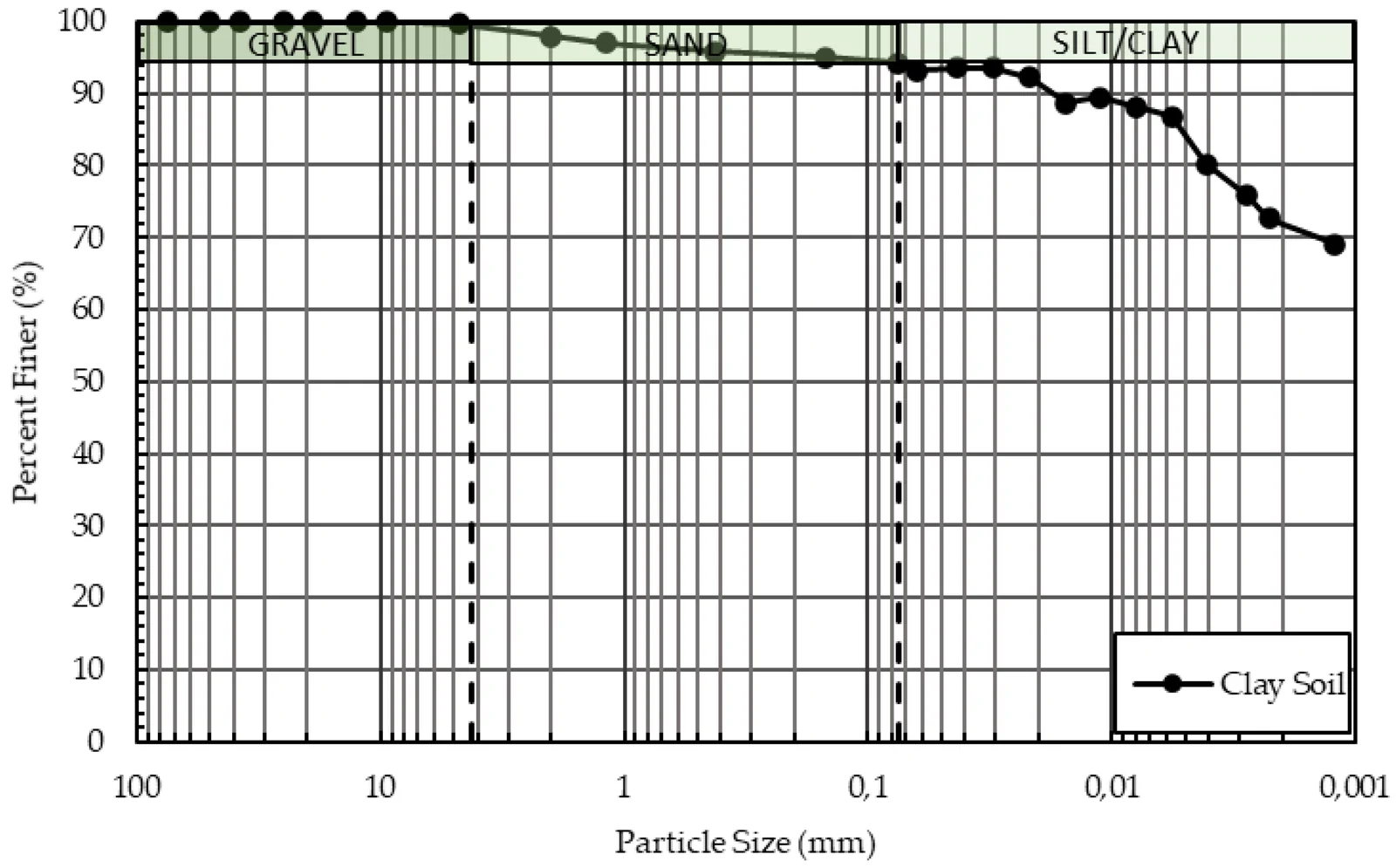
Particle-size distribution curve of the clay soil used in this study.

The particle-size distribution curve of the soil is presented in Figure 3. Grain-size analysis performed in accordance with ASTM D6913 [29] indicated that approximately 93% of the material passed the No. 200 sieve (0.075 mm), confirming its predominantly fine-grained nature. Atterberg limit tests conducted according to ASTM D4318 [30] yielded a liquid limit (LL) of 128%, a plastic limit (PL) of 31%, and a plasticity index (PI) of 97%, indicating extremely high plasticity. Based on the Unified Soil Classification System ASTM D2487 [31], the soil was classified as CH (high-plasticity clay), whereas according to AASHTO M145 [32], it was categorized as A-7-5.

Standard Proctor compaction tests performed in accordance with ASTM D698 [33] yielded a maximum dry unit weight of 16.85 kN/m^3^ and an optimum moisture content of 32.3%. These characteristics demonstrate the high-water affinity, pronounced compressibility, and moisture-sensitive mechanical behavior of the tested clay, making it an appropriate material for evaluating the stabilization effects of PVC powder and recycled polyester fibers.

#### 2.1.2. PVC Powder

The PVC powder used in this study was supplied by Pidosan Plastik Doğrama ve İnşaat San. A.Ş (Ankara, Turkey). The material is a white polyvinyl chloride (PVC) powder produced by the suspension polymerization process with a K-value of 68, corresponding to a medium molecular weight resin. The bulk density of the PVC powder was 0.56 g/cm^3^, providing good flowability and facilitating homogeneous mixing with the clay soil. Particle-size characterization of the PVC powder showed that approximately 94% of the material passed the 0.250-mm sieve, with the particles predominantly ranging between 0.63 and 0.250 mm. The relatively fine particle size of the PVC powder facilitated its distribution within the clay matrix and allowed the particles to occupy part of the interaggregate void space, thereby contributing to the physical void-filling and particle-rearrangement mechanisms observed in the SEM analysis.

Owing to its stable physical properties and uniform particle morphology, the material was selected for evaluating the influence of PVC incorporation on the engineering behavior of high-plasticity clay.

#### 2.1.3. Recycled Polyester Fiber

The reinforcing material used in this study consisted of 100% regenerated polyester staple fibers manufactured from recycled polyethylene terephthalate (PET) and textile wastes. The fibers were supplied by Ata Fiber Industry and exhibited a natural semi-dull appearance with a fineness of 6 denier and an initial cut length of 64 mm. To ensure a more uniform distribution within the soil matrix and to minimize fiber agglomeration during specimen preparation, the fibers were manually cut into smaller lengths before mixing. The initial 64 mm fibers were manually cut in two sequential stages. First, each fiber was divided approximately into two equal parts, and the resulting segments were subsequently divided once more into approximately equal halves, yielding a nominal final fiber length of approximately 16 mm. No sieving or additional mechanical size-classification procedure was applied after cutting. The sequential cutting procedure was adopted to obtain a relatively consistent fiber size while facilitating fiber separation and uniform dispersion within the clay matrix. The fibers were of the solid/hollow conjugated type and possessed a silicone finish with flame-retardant properties. In addition to providing mechanical reinforcement, the use of recycled polyester fibers promotes the sustainable reuse of post-consumer plastic and textile wastes. According to the material characterization data provided by the manufacturer, the recycled polyester fibers had a linear density of 6.80 denier and a tenacity of 3.25 cN/dtex, with an elongation at break of 65%. The fibers had an initial length of 64 mm and a moisture content of 0.51%. The manufacturer did not provide a directly measured Young’s modulus for the recycled polyester fibers; therefore, an experimentally determined elastic modulus is not reported in the present study. Nevertheless, the reported tenacity and elongation characteristics indicate that the fibers possess sufficient tensile resistance and deformation capacity to act as discrete reinforcement within the compacted clay matrix. These mechanical characteristics are relevant to the reinforcement mechanism, as the tensile resistance and deformability of the fibers facilitate stress transfer within the soil–fiber system, restrict relative particle displacement, and promote crack-bridging under axial loading.

### 2.2. Methods

#### 2.2.1. Mix Design and Specimen Preparation

Based on the geotechnical characterization of the natural clay soil, an experimental program was designed to investigate the effects of PVC powder and recycled polyester fibers on the engineering behavior of high-plasticity clay. The untreated clay was used as the control material, whereas PVC powder and recycled polyester fibers were incorporated separately at different dosage levels selected based on previous studies reported in the literature. PVC powder was incorporated at 5%, 10%, and 15% by dry weight of the soil. These dosage levels were selected to represent low, intermediate, and relatively high PVC contents and are consistent with the range investigated in previous studies on PVC-modified clayey soils [22,23,24,25]. Recycled polyester fibers were incorporated at 0.5%, 0.75%, and 1.0% by dry weight of the soil. This relatively low fiber-content range was selected considering previous experimental studies [22] showing that the mechanical response of fiber-reinforced soils is strongly dependent on fiber dosage and that effective reinforcement can be achieved at relatively low polyester fiber contents. In addition, following the evaluation of the individual compaction responses, 5% PVC powder and 0.5% recycled polyester fiber exhibited the most favorable compaction characteristics within their respective dosage ranges and were therefore selected for the hybrid mixture. The purpose of this hybrid composition was to investigate whether combining the favorable low-content levels of the two recycled additives could provide complementary effects on compaction, strength, deformation behavior, and microstructural characteristics. Accordingly, the hybrid mixture was designed as a representative combined formulation rather than as a comprehensive optimization of all possible PVC–fiber dosage combinations. In this context, complementary functionality refers to the combined contribution of PVC-induced modification of particle packing and fiber-induced reinforcement, rather than a super-additive increase in strength. Therefore, the results obtained for this mixture are interpreted specifically for the investigated 5% PVC + 0.5% fiber combination and should not be regarded as establishing a generalized optimum or universal complementary effects relationship for the hybrid system. Accordingly, the selected dosage levels were intended to provide a systematic assessment of the response of the clay to increasing additive contents while remaining within ranges previously investigated for polymer-modified soils. The hybrid dosage (5% PVC + 0.5% polyester fiber) was selected based on the most favorable compaction responses obtained at the lower individual additive contents, rather than as a predetermined optimum combination. The mixture design adopted in this study is summarized in Table 2.

The direct addition method was adopted throughout the specimen preparation process; therefore, the additives were incorporated into the natural clay without replacing any portion of the soil. All additive contents were expressed as percentages of the dry weight of the soil. Prior to mixing, the clay soil was air-dried, pulverized, and thoroughly homogenized. The required amount of PVC powder was first blended with the dry soil to ensure a uniform distribution throughout the soil matrix. For the fiber-reinforced mixtures, the polyester fibers were manually separated before mixing to minimize fiber agglomeration and improve their dispersion within the soil. Distilled water was then gradually added until the target moisture content was reached, followed by continuous mixing to obtain a homogeneous consistency. Finally, the prepared mixtures were sealed in airtight polyethylene bags and stored for 24 h at approximately 20–25 °C under laboratory conditions. Relative humidity was not specifically controlled; however, the use of airtight polyethylene bags was intended to minimize moisture exchange with the surrounding environment and facilitate moisture redistribution within the clay matrix.

#### 2.2.2. Standard Proctor Compaction Test

The compaction characteristics of the untreated and modified soils were determined using the Standard Proctor compaction test in accordance with ASTM D698 [27]. The optimum moisture content (OMC) and maximum dry unit weight (MDD) obtained from the compaction tests were subsequently used for preparing all specimens employed in the mechanical and microstructural investigations.

For the untreated clay, constant air-void lines corresponding to *V_A_* = 0%, 5%, and 10% were calculated and plotted together with the compaction curve to provide theoretical reference boundaries for interpreting the relationship between moisture content and dry unit weight. The dry unit weight corresponding to a specified air-void content was calculated from the soil phase relationships as:

$$\gamma_{d}=\frac{G_{s}\gamma_{w}(1-V_{A})}{1+wG_{s}}$$

#### 2.2.3. Unconfined Compressive Strength Test

The unconfined compressive strength (UCS) tests were performed in accordance with ASTM D2166 [34] to evaluate the effects of PVC powder and recycled polyester fiber on the strength behavior of the high-plasticity clay. Cylindrical specimens were prepared at the corresponding optimum moisture content and maximum dry unit weight determined from the Standard Proctor compaction tests. After compaction, the specimens were carefully extruded from the molds, sealed to minimize moisture exchange, and conditioned for 24 h under the same laboratory conditions prior to testing. The 24 h conditioning period was adopted as a consistent specimen-preparation interval to facilitate moisture redistribution within the compacted specimens while maintaining identical conditioning conditions for all mixtures. This period was not intended to represent a chemical curing process, since the recycled PVC powder and polyester fibers used in this study are non-cementitious physical modifiers and no chemically reactive binder was incorporated. Accordingly, the reported UCS values should be interpreted as the short-term mechanical response of the modified clay under a standardized 24 h conditioning period rather than as long-term or fully equilibrated strength values.

For each mixture, three replicate specimens were prepared and tested under identical conditions, and the average UCS value was used to represent the compressive strength of the corresponding mixture.

**Figure 4:**
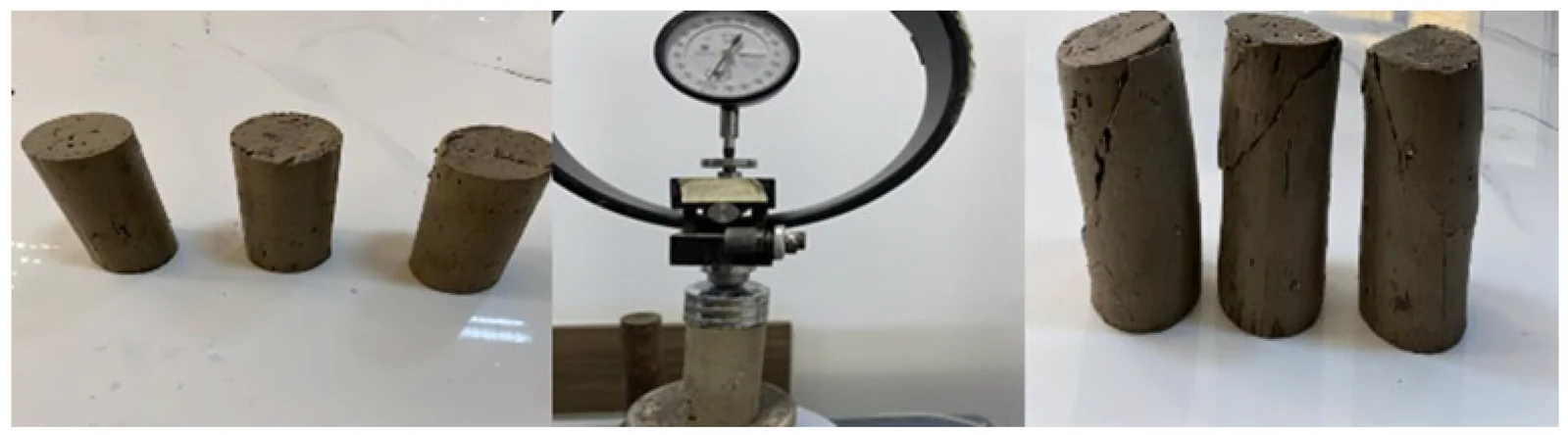
Representative cylindrical specimens prepared for the UCS tests.

The UCS tests were carried out using a computer-controlled compression testing machine under displacement-controlled loading at a constant strain rate specified by ASTM D2166 [34]. During testing, the applied load and axial deformation were continuously recorded until specimen failure, and the peak compressive stress was reported as the unconfined compressive strength. Representative cylindrical specimens prepared for the UCS tests are shown in Figure 4.

#### 2.2.4. Scanning Electron Microscopy (SEM) Analysis

The microstructural characteristics of the untreated and modified clay specimens were investigated using a Hitachi SU5000 scanning electron microscope (Hitachi, Tokyo, Japan) at the Central Research Laboratory (MERLAB), Ankara Yıldırım Beyazıt University, Ankara, Türkiye. Prior to SEM examination, representative specimens were oven-dried and coated with a thin conductive gold (Au) layer to minimize surface charging during imaging. Secondary electron (SE) mode was employed for image acquisition. The SEM observations were performed at an accelerating voltage of 5 kV with a working distance of approximately 10.8 mm. Representative micrographs were obtained at different magnification levels corresponding to 50, 10, and 2 μm scale bars to evaluate both the overall morphology and the detailed microstructural characteristics of the untreated and stabilized clay specimens.

#### 2.2.5. X-Ray Diffraction (XRD)

The mineralogical characteristics of the untreated clay and the selected stabilized mixtures were investigated by X-ray diffraction (XRD) analysis at the Central Research Laboratory (MERLAB), Ankara Yıldırım Beyazıt University, Ankara, Türkiye. Diffraction patterns were collected over a 2θ range of 10–90° at a scanning speed of 20° min^−1^. The obtained diffraction patterns were used to identify the major crystalline mineral phases present in the untreated clay and to evaluate possible mineralogical changes after the incorporation of PVC powder and recycled polyester fiber.

## 3. Results and Discussion

### 3.1. Compaction Characteristics

**Figure 5:**
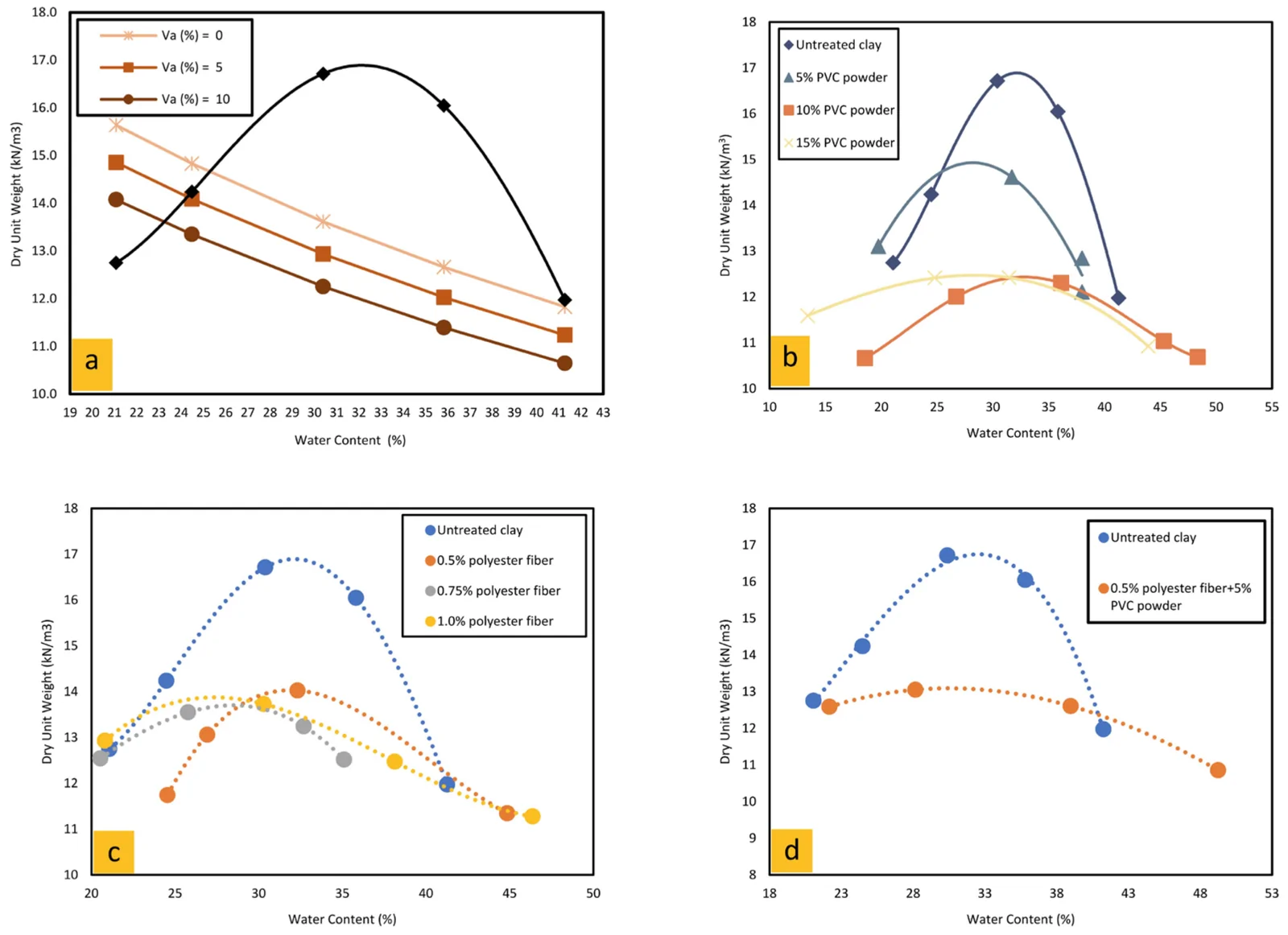
Compaction characteristics of the untreated and modified clay mixtures: (**a**) compaction curve and constant air-void lines of the untreated clay, (**b**) effect of PVC powder content, compaction curve of the untreated clay (black line) and constant air-void lines (**c**) effect of polyester fiber content, and (**d**) comparison of the untreated clay and the hybrid mixture containing 5% PVC powder and 0.5% polyester fiber.

The compaction characteristics of the untreated high-plasticity clay were evaluated to establish a reference condition for assessing the effects of PVC powder and recycled polyester fiber incorporation. As shown in Figure 5a, the untreated clay exhibited the typical bell-shaped compaction curve characteristic of fine-grained soils, in which the dry unit weight increased with increasing moisture content up to the optimum condition and subsequently decreased with further water addition. The untreated clay achieved a maximum dry unit weight of 16.85 kN/m^3^ at an optimum moisture content of 32.3%. The relatively high optimum moisture content is consistent with the high plasticity of the soil (LL = 128% and PI = 97%), reflecting the large amount of water required to facilitate particle rearrangement during compaction. The constant air-void lines presented in Figure 5a provide theoretical reference boundaries for interpreting the compaction state of the untreated clay.

The incorporation of PVC powder modified the compaction response of the clay, as illustrated in Figure 5b. All PVC-treated mixtures exhibited lower maximum dry unit weights than the untreated clay, although the magnitude of the reduction varied with PVC content. Among the investigated mixtures, the specimen containing 5% PVC powder exhibited the highest maximum dry unit weight, whereas increasing the PVC content to 10% and 15% resulted in progressively lower dry unit weights. This trend is associated with the relatively low density of PVC compared with the mineral soil particles and the resulting changes in the internal packing arrangement of the compacted matrix. Consequently, increasing the PVC content reduced the overall dry unit weight, while a limited addition of 5% PVC maintained a comparatively more favorable compaction response.

The incorporation of recycled polyester fibers also influenced the compaction behavior of the clay, as shown in Figure 5c. All fiber-reinforced mixtures exhibited lower maximum dry unit weights than the untreated clay. Among the investigated fiber contents, the mixture containing 0.5% recycled polyester fiber produced the highest maximum dry unit weight, whereas further increases to 0.75% and 1.0% resulted in lower values. The results indicate that increasing the fiber content progressively affected the packing efficiency of the compacted soil matrix, leading to a reduction in dry unit weight.

Based on the individual compaction responses, 5% PVC powder and 0.5% recycled polyester fiber exhibited the most favorable compaction characteristics within their respective dosage ranges and were therefore selected for preparing the hybrid mixture. As presented in Figure 5d, the hybrid mixture exhibited an optimum moisture content of approximately 31.5% and a maximum dry unit weight of 13.10 kN/m^3^. Compared with the untreated clay, the maximum dry unit weight decreased by approximately 22.3%, whereas the optimum moisture content decreased only slightly (2.5%). These results indicate that the combined incorporation of PVC powder and recycled polyester fiber influenced the dry density of the compacted soil to a much greater extent than its moisture requirement during compaction.

Within the investigated hybrid mixture, PVC powder is considered to have the more pronounced influence on the compaction response because of its substantially higher dosage (5%) compared with the polyester fiber content (0.5%) and its direct contribution to the replacement of mineral soil contacts by lower-density polymer particles. The polyester fibers additionally disrupt particle rearrangement and packing through their discrete fibrous geometry. Therefore, the marked reduction in maximum dry unit weight of the hybrid mixture is interpreted as being primarily associated with PVC incorporation, with the fibers providing an additional packing-disruption effect. However, since only one hybrid dosage was investigated, this interpretation should not be generalized to other PVC–fiber proportions.

Overall, the compaction results demonstrate that both PVC powder and recycled polyester fiber modify the compaction characteristics of high-plasticity clay by reducing the maximum dry unit weight while producing only limited changes in optimum moisture content. The mixtures containing 5% PVC powder and 0.5% recycled polyester fiber exhibited the most favorable compaction responses among the investigated dosage levels and were therefore selected for the preparation of the hybrid mixture and the subsequent mechanical and microstructural investigations.

### 3.2. Unconfined Compressive Strength

**Figure 6:**
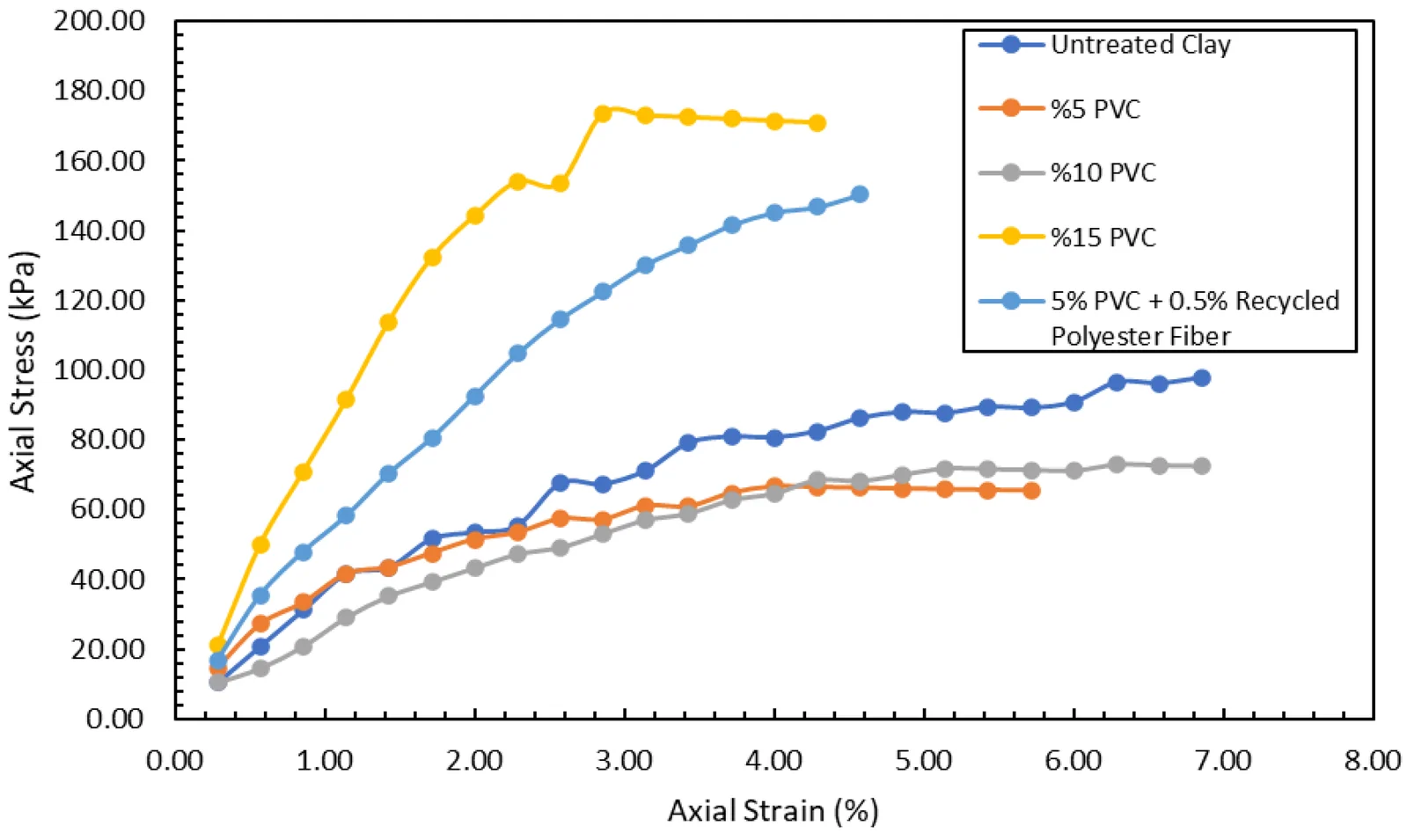
Axial stress–strain curves of untreated clay and PVC-modified clay specimens.

**Figure 7:**
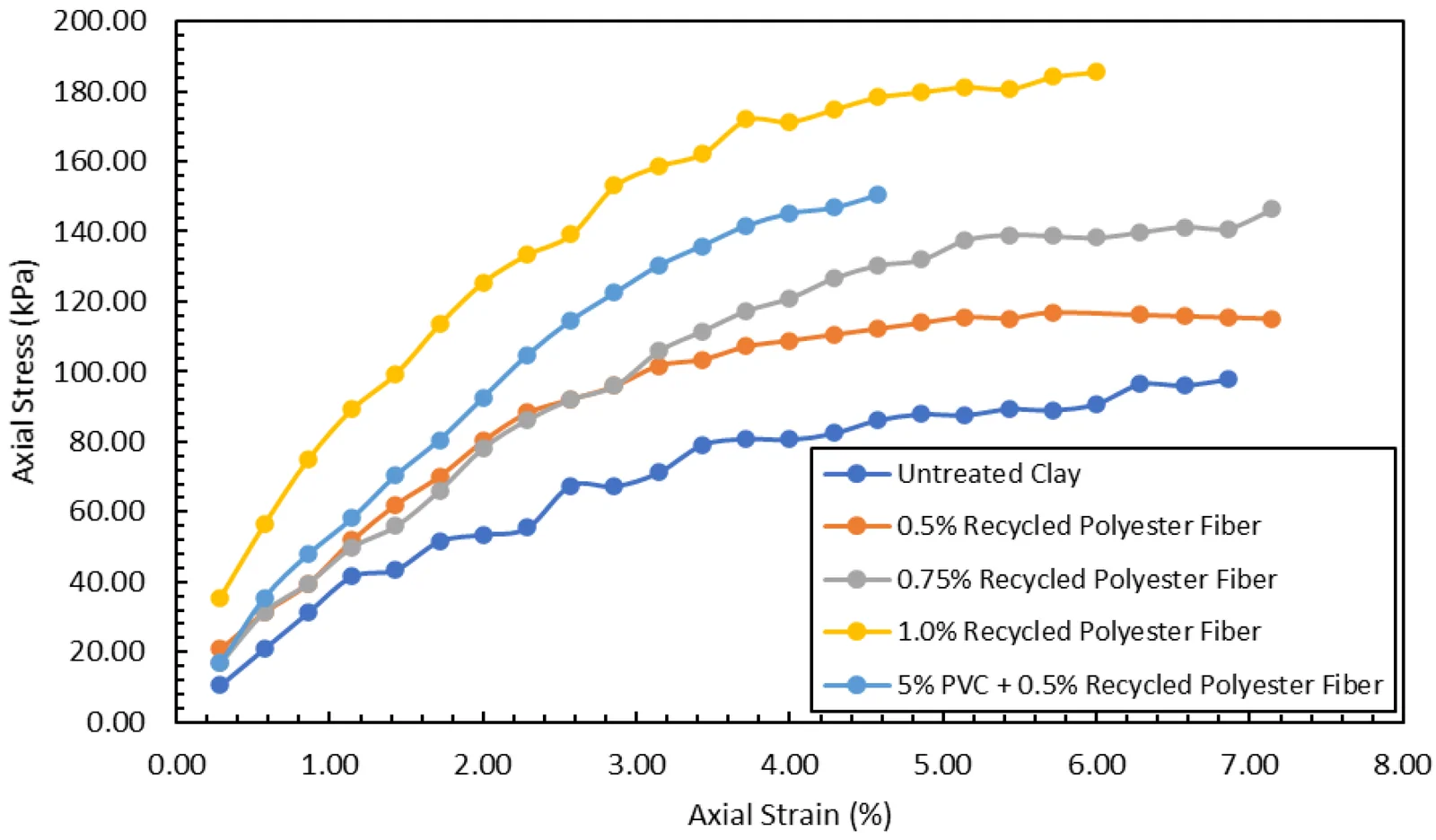
Axial stress–strain curves of untreated clay and recycled polyester fiber-reinforced specimens.

**Figure 8:**
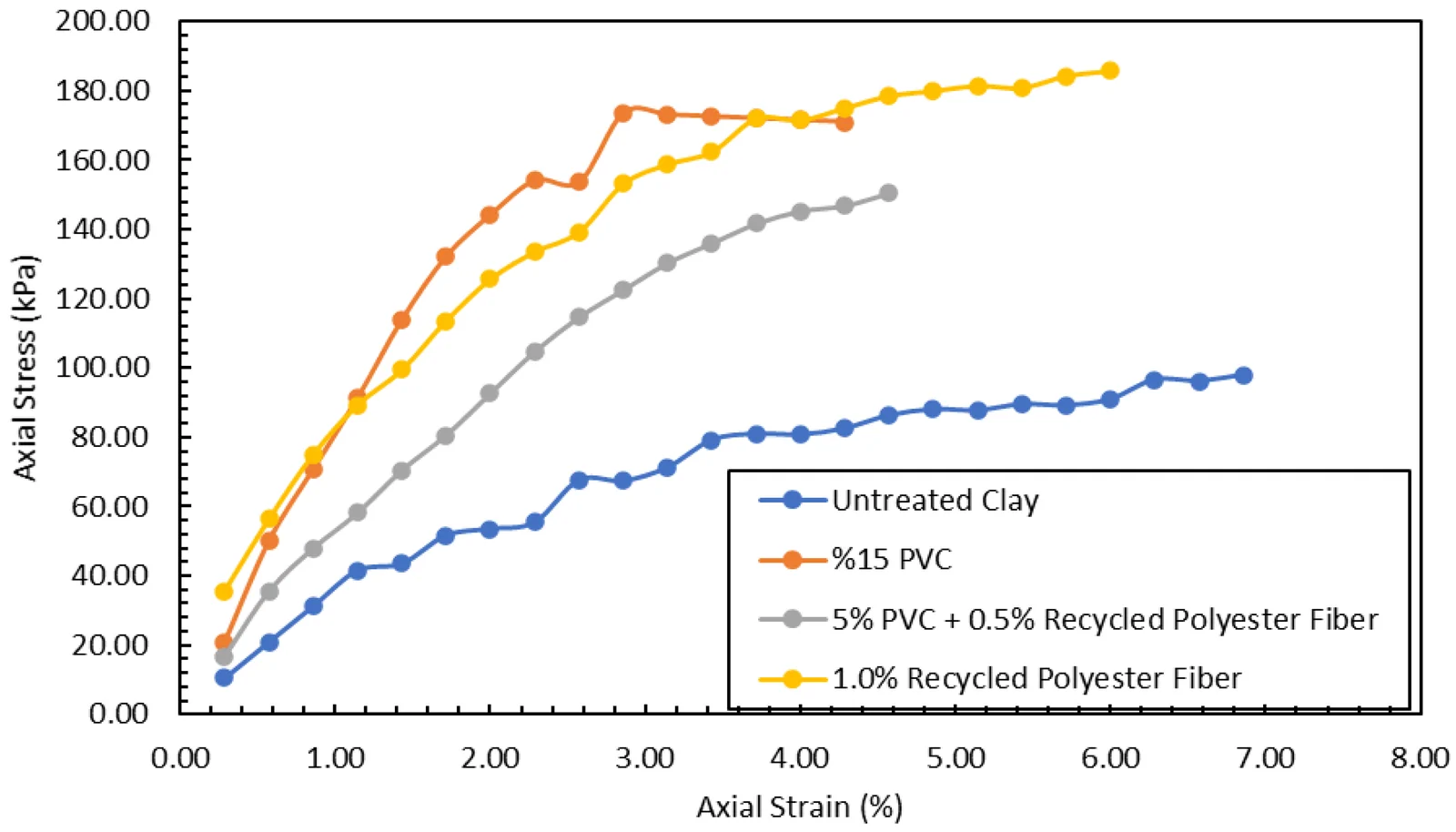
Comparison of the axial stress–strain responses of untreated clay and the selected high-performing modified mixtures.

The unconfined compressive strength (UCS) tests were conducted to evaluate the effects of PVC powder, recycled polyester fiber, and their combined incorporation on the mechanical behavior of the high-plasticity clay. The axial stress–strain responses of the untreated and modified specimens are presented in Figure 6, Figure 7 and Figure 8.

The stress–strain responses of the untreated clay and the PVC-modified mixtures are presented in Figure 6. The untreated clay exhibited a gradual increase in axial stress, reaching a peak compressive strength of 97.84 kPa at an axial strain of 6.86%. The addition of 5% and 10% PVC powder reduced the peak compressive strengths to 72.62 kPa and 75.04 kPa, corresponding to decreases of approximately 25.8% and 23.3%, respectively, compared with the untreated clay. In contrast, the specimen containing 15% PVC powder exhibited the highest compressive strength among the PVC-modified mixtures, reaching 204.08 kPa, which represents an increase of approximately 108.6% relative to the untreated specimen. The corresponding stress–strain curve also exhibited a steeper initial slope, indicating increased stiffness during the early stages of loading. However, failure occurred at a lower axial strain (2.86%), indicating a reduction in deformation capacity compared with the untreated clay.

The non-monotonic variation in UCS with increasing PVC content can be interpreted by considering the combined effects of compaction behavior, particle interaction, and microstructural modification. At relatively low PVC contents (5% and 10%), the incorporation of low-density polymer particles reduced the maximum dry unit weight relative to the untreated clay and altered the original clay–clay contact network. At these dosage levels, the PVC particles may have acted predominantly as discrete inclusions within the soil matrix, partially interrupting direct contacts between clay aggregates without providing a sufficiently extensive filling or rearrangement effect to compensate for the resulting reduction in soil-particle interaction. This interpretation is consistent with the observed reductions in UCS of 25.8% and 23.3% for the 5% and 10% PVC mixtures, respectively. In contrast, at 15% PVC content, the substantially greater amount of polymer particles appears to have produced a different internal packing condition, allowing more extensive redistribution of particle contacts and greater participation of PVC particles within the load-transfer network. This transition may explain the pronounced increase in UCS observed for the 15% PVC mixture. However, its lower failure strain indicates that the resulting structure was stronger but more brittle than the untreated clay. Therefore, the observed response suggests a dosage-dependent transition in the role of PVC particles rather than a monotonic relationship between PVC content and strength.

The stress–strain responses of the recycled polyester fiber-reinforced specimens are presented in Figure 7. The compressive strength increased progressively with increasing fiber content. The UCS values increased from 117.04 kPa for the 0.5% fiber mixture to 146.31 kPa and 185.63 kPa for the 0.75% and 1.0% fiber mixtures, corresponding to strength improvements of approximately 19.6%, 49.5%, and 89.7%, respectively, compared with the untreated clay. The fiber-reinforced specimens also maintained relatively high deformation capacities. The mixtures containing 0.5% and 0.75% fiber reached failure strains of approximately 7.14%, whereas the specimen containing 1.0% fiber failed at an axial strain of 6.00% while exhibiting the highest compressive strength among the fiber-reinforced specimens. The stress–strain curves showed a gradual increase in stress without abrupt post-peak failure, indicating that fiber reinforcement enhanced both strength and deformation capacity.

A comparison of the untreated clay with the selected high-performance mixtures is presented in Figure 8. Among all mixtures, the specimen containing 15% PVC powder exhibited the highest peak compressive strength (204.08 kPa), whereas the specimen reinforced with 1.0% recycled polyester fiber reached a slightly lower peak stress (185.63 kPa) while maintaining a comparatively greater deformation capacity. The hybrid mixture containing 5% PVC powder and 0.5% recycled polyester fiber achieved a peak compressive strength of 150.36 kPa, corresponding to an increase of approximately 53.7% compared with the untreated clay. Although the hybrid mixture did not reach the maximum strength obtained with the optimum individual PVC or fiber mixtures, it exhibited a balanced stress–strain response by combining a substantial increase in compressive strength with moderate deformation capacity.

Accordingly, the hybrid response should not be interpreted as evidence of a super-additive synergistic strength gain. Rather, it demonstrates complementary functionality, in which PVC-induced particle rearrangement and fiber-induced reinforcement contribute to a balanced combination of strength and deformation capacity.

The mechanical response of the hybrid mixture further indicates that the combined incorporation of PVC powder and polyester fiber should not be interpreted as producing a synergistic or super-additive increase in peak strength. Although the 5% PVC + 0.5% fiber mixture increased the UCS by 53.7% relative to the untreated clay, its peak strength remained lower than those obtained with 15% PVC and 1.0% fiber individually. The significance of the investigated hybrid composition therefore lies primarily in its balanced mechanical response and the simultaneous mobilization of two different modification mechanisms: PVC-induced changes in particle packing and fiber-induced tensile restraint and bridging. Since only one combined dosage was examined, the present results demonstrate the feasibility and complementary behavior of the selected hybrid composition but do not establish an optimum PVC–fiber ratio or a generalized dosage-dependent complementary effects relationship.

The combined improvement in strength and deformation behavior observed in the hybrid mixtures is consistent with previous investigations on fiber-reinforced clay soils [26].

It should be noted that the UCS results obtained in the present study represent the short-term mechanical response after a 24 h conditioning period. Previous investigations involving chemically active or aqueous polymer stabilizers have demonstrated that curing duration may substantially influence strength development, particularly over 7- and 28-day periods. However, the recycled PVC powder and polyester fibers investigated here were used as non-cementitious physical modifiers, and the stabilization mechanisms identified by the SEM and XRD analyses were predominantly associated with particle rearrangement, physical void filling, fiber bridging, and mechanical interlocking rather than the formation of new reaction products. Therefore, the present results should not be interpreted as demonstrating long-term strength development. Additional UCS testing at extended conditioning periods, including 7 and 28 days, would be required to determine whether time-dependent changes occur in the mechanical performance of these recycled polymer-modified clay mixtures.

### 3.3. SEM Analysis

**Figure 9:**
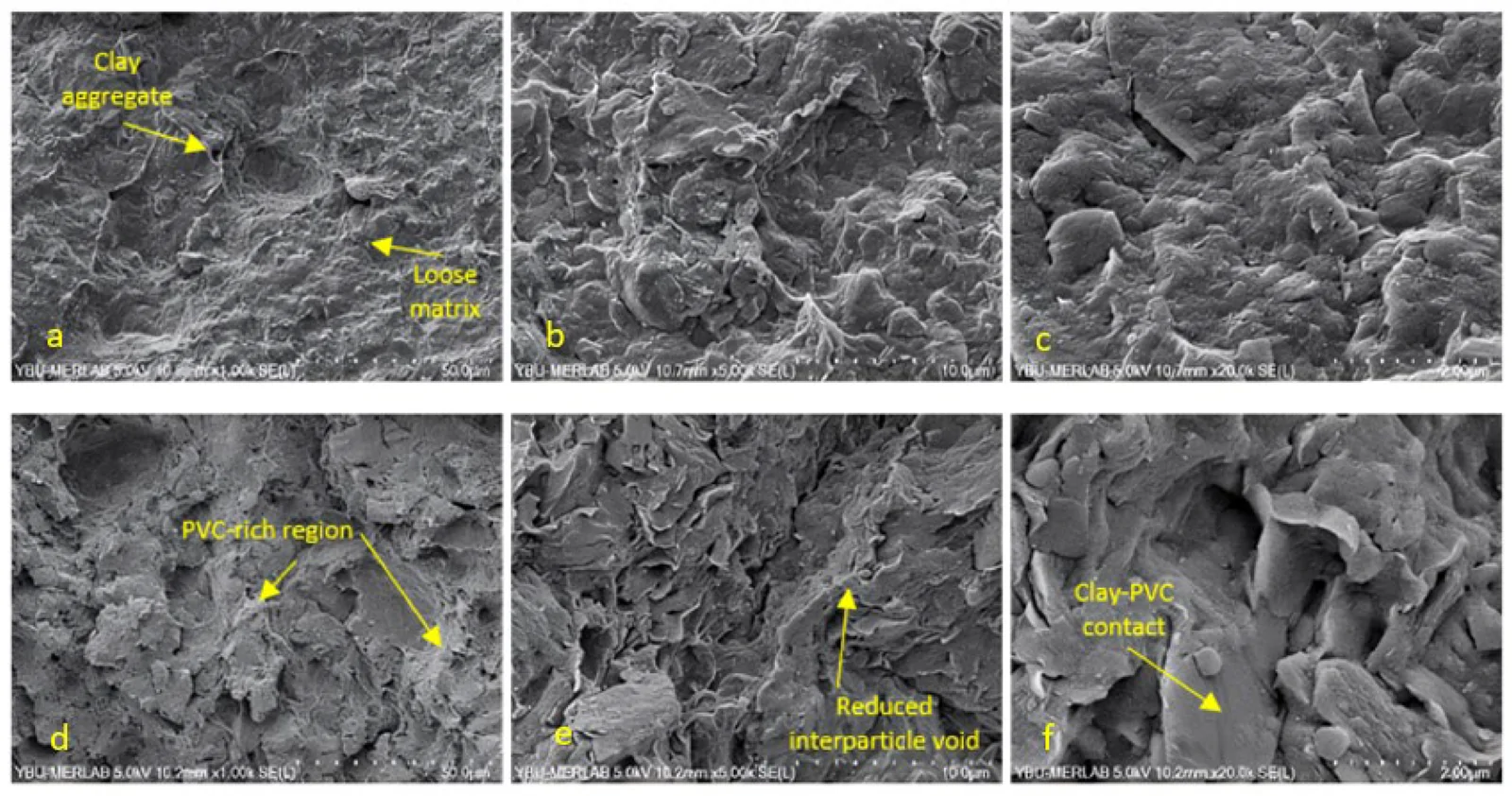
SEM micrographs of (**a**–**c**) untreated clay and (**d**–**f**) clay containing 10% PVC powder at different magnification levels. The labelled features highlight representative clay aggregates, interaggregate voids, PVC particles, and modified particle-contact regions.

**Figure 10:**
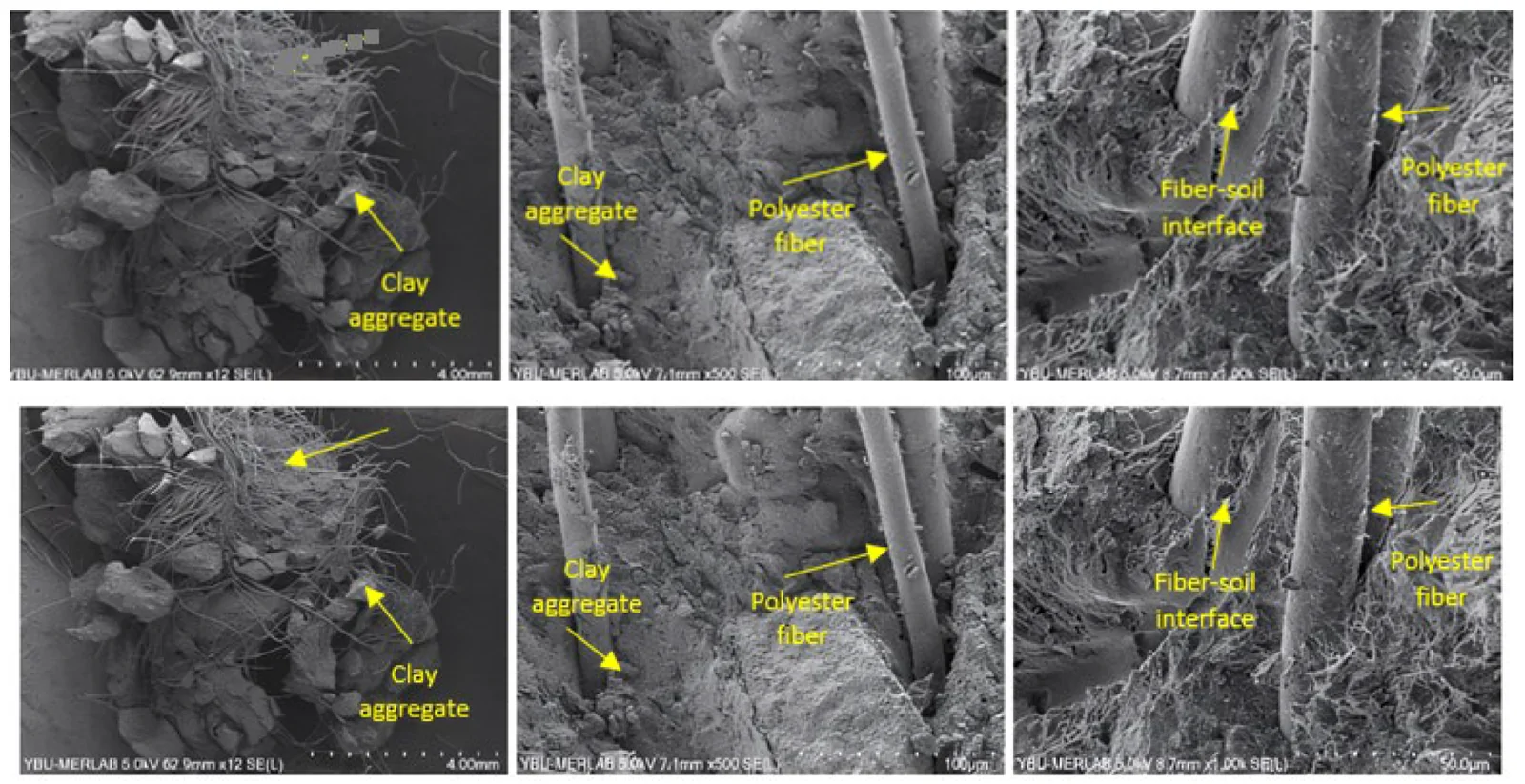
SEM micrographs of clay reinforced with 0.5% recycled polyester fiber at different magnifications, illustrating the distribution of polyester fibers, fiber–soil interaction, and the formation of a three-dimensional reinforcing network.

**Figure 11:**
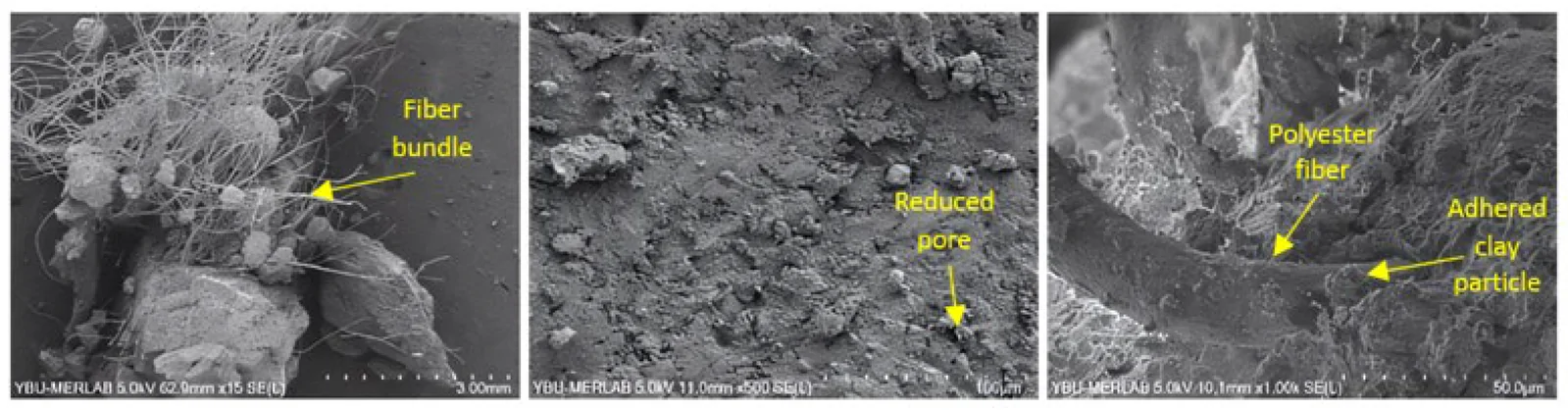
SEM micrographs of the hybrid specimen containing 5% PVC powder and 0.5% recycled polyester fiber at different magnifications, illustrating fiber distribution, reduced pore continuity, clay–fiber interaction, and the combined microstructural effects of PVC powder and recycled polyester fiber.

SEM analysis was conducted to investigate the microstructural modifications induced by the incorporation of PVC powder and recycled polyester fibers into the high-plasticity clay. Representative SEM micrographs of the untreated clay and the selected modified mixtures are presented in Figure 9, Figure 10 and Figure 11. The observations were used to evaluate changes in particle arrangement, pore distribution, interparticle contacts, and the interaction between the clay matrix and the incorporated additives.

The specimens selected for SEM examination were intended to provide representative microstructural evidence of the principal soil–additive interaction mechanisms rather than to establish a direct microstructure–strength correlation for the highest-performing mixtures. Accordingly, the SEM observations were used primarily to identify changes in particle arrangement, pore characteristics, fiber–soil interaction, and the complementary physical roles of the recycled additives.

Figure 9 presents SEM micrographs of the untreated clay (upper row) and the clay containing 10% PVC powder (lower row) at different magnifications. The untreated clay exhibited a heterogeneous and relatively open microstructure composed of irregular clay aggregates, discontinuous interparticle contacts, and visible interaggregate voids. The clay particles appeared loosely packed with a flocculated arrangement, indicating a nonuniform internal fabric and limited particle interlocking. Localized pores and microcracks were also evident within the untreated matrix.

As indicated by the labels in Figure 9, the untreated clay exhibits distinct clay aggregates separated by visible interaggregate voids. Following PVC incorporation, PVC particles are distributed within the clay matrix and locally modify the arrangement and contact conditions of the surrounding soil particles. These observations support the interpretation that PVC primarily acts through physical particle rearrangement and partial occupation of available void spaces rather than through the formation of a new cementitious phase.

In contrast, the specimen containing 10% PVC powder exhibited a denser and more continuous microstructure. The polymer particles were distributed within the clay matrix and occupied part of the interaggregate void space, resulting in reduced pore continuity and closer contact between adjacent clay particles. Smooth polymer-rich regions and clay–PVC interfaces were observed, indicating that PVC acted primarily as a physical inclusion rather than forming cementitious bonds. The reduced interparticle voids and the more compact particle arrangement suggest that the primary stabilization mechanism involved physical void filling and particle rearrangement. Overall, the incorporation of 10% PVC powder produced a more compact internal fabric than the untreated clay.

Figure 10 presents SEM micrographs of the clay reinforced with 0.5% recycled polyester fiber at different magnifications. The low-magnification image shows that the recycled polyester fibers were distributed throughout the clay matrix, forming a three-dimensional interconnected network linking adjacent clay aggregates. At higher magnifications, the fibers were observed to be firmly embedded within the clay matrix, with numerous clay particles adhering to their surfaces. The close fiber–soil contact and the presence of adhered clay particles indicate effective mechanical interlocking and interfacial friction between the fibers and the surrounding soil. Several fibers bridged adjacent clay aggregates, restricting their relative movement and improving the continuity of the soil skeleton. No evidence of chemical bonding was observed, indicating that the reinforcing effect of the recycled polyester fibers was governed primarily by mechanical mechanisms, including fiber bridging, interfacial friction, and particle interlocking. These observations are consistent with the improved strength and deformation characteristics obtained from the UCS tests.

Figure 11 presents SEM micrographs of the hybrid specimen containing 5% PVC powder and 0.5% recycled polyester fiber at different magnifications. The recycled polyester fibers were uniformly distributed throughout the clay matrix, forming an interconnected three-dimensional reinforcing network. At higher magnifications, the hybrid specimen exhibited a denser and more homogeneous microstructure than the untreated clay, characterized by reduced pore continuity and improved particle packing. Clay particles were firmly adhered to the fiber surfaces, indicating intimate fiber–soil contact and effective mechanical interlocking at the interface. The simultaneous presence of a compact soil matrix and embedded reinforcing fibers demonstrates the complementary roles of the two stabilizing materials. PVC powder primarily reduced pore continuity through a physical filling effect, whereas the recycled polyester fibers provided bridging and tensile restraint between adjacent soil aggregates. Consequently, the hybrid specimen developed a more integrated microstructure than the untreated clay, reflecting the combined effects of PVC-induced densification and fiber reinforcement. These observations provide a microstructural explanation for the improved mechanical behavior of the hybrid mixture observed in the UCS tests.

Although the SEM observations provide useful evidence of the physical interaction mechanisms associated with PVC powder and polyester fiber incorporation, the 15% PVC and 1.0% fiber specimens, which exhibited the highest UCS values within their respective groups, were not examined by SEM. Therefore, the present microstructural observations should not be interpreted as direct evidence of the mechanisms responsible for the maximum UCS values. Instead, they support the general mechanisms of PVC-induced particle rearrangement and void filling and fiber-induced bridging and mechanical interlocking. A direct microstructure–strength correlation for the highest-performing mixtures requires further microstructural investigation.

The SEM observations presented in this study provide qualitative evidence of particle rearrangement, local pore occupation, and fiber-bridging mechanisms. However, quantitative image-analysis parameters, such as pore-area fraction, fiber orientation, and soil–fiber interface width, were not determined. Therefore, direct quantitative correlations between these microstructural features and macroscopic parameters such as UCS, failure strain, or stiffness cannot be established from the present dataset. Future studies incorporating standardized SEM image analysis and a larger number of representative micrographs would enable a more rigorous micro–macro correlation.

### 3.4. X-Ray Diffraction (XRD) Analysis

XRD analysis was performed to evaluate the mineralogical composition of the untreated and treated clay specimens and to determine whether the incorporation of PVC powder and polyester fiber resulted in detectable changes in the crystalline phases. The identified crystalline phases and the main XRD observations are summarized in Table 3.

As summarized in Table 3, the untreated clay was characterized by illite, quartz, kaolinite, and calcite. The same principal crystalline phases were identified in the specimens containing 5% PVC, 0.5% fiber, and the hybrid combination of 5% PVC and 0.5% fiber, indicating that the principal mineralogical composition of the clay was retained after the incorporation of the recycled additives.

Comparison of the diffraction results showed that the incorporation of PVC powder and polyester fiber did not result in the identification of distinct new crystalline phases. Furthermore, the characteristic diffraction positions of the principal mineral phases were generally retained after treatment. For example, the characteristic calcite reflection occurred at approximately 29.42° 2θ in the untreated clay and remained at approximately 29.41–29.44° 2θ in the treated specimens. These observations indicate that the original crystalline mineralogical structure of the clay was largely preserved following the incorporation of the recycled additives. This mineralogical stability is an important finding because it indicates that the improvements observed in mechanical behavior cannot be attributed to the formation of new crystalline reaction products. Instead, the XRD results provide independent evidence supporting the predominantly physical nature of the stabilization mechanism.

When considered together with the SEM observations, the results suggest that PVC particles primarily contributed to changes in particle arrangement and void filling, whereas the polyester fibers provided mechanical bridging and interfacial interaction within the soil matrix. The hybrid system therefore appears to modify the soil structure mainly through the complementary physical effects of PVC-induced packing and fiber reinforcement rather than through substantial mineralogical transformation. Thus, XRD complements the SEM and UCS findings by distinguishing physical soil-fabric modification and mechanical reinforcement from mineralogical transformation as the principal mechanisms governing the observed response.

However, because only one hybrid composition was examined, the present results should be interpreted as evidence of the feasibility and complementary behavior of the selected 5% PVC + 0.5% fiber mixture rather than as identification of an optimum hybrid dosage or a generalized synergistic relationship. A broader factorial investigation incorporating multiple PVC–fiber combinations is required to establish the dosage-dependent interaction and optimize the hybrid stabilization system.

## 4. Conclusions

This study investigated the effects of PVC powder and recycled polyester fibers, used individually and in combination, on the compaction behavior, unconfined compressive strength, and microstructural characteristics of a high-plasticity clay. Based on the experimental findings, the following conclusions can be drawn:

1. The incorporation of PVC powder and recycled polyester fibers altered the compaction characteristics of the high-plasticity clay by reducing the maximum dry unit weight relative to the untreated soil. Among the investigated mixtures, 5% PVC powder and 0.5% recycled polyester fiber exhibited the most favorable compaction responses and were therefore selected for preparing the hybrid mixture.
2. The mechanical response of the clay was strongly dependent on the type and content of the incorporated additive. The specimen containing 15% PVC powder achieved the highest unconfined compressive strength (204.08 kPa), corresponding to an increase of approximately 108.6% compared with the untreated clay, although this improvement was accompanied by reduced deformation capacity.
3. Recycled polyester fiber provided simultaneous improvements in strength and deformation behavior. The specimen reinforced with 1.0% recycled polyester fiber exhibited an approximately 89.7% increase in compressive strength while maintaining a relatively high failure strain, demonstrating the effectiveness of fiber reinforcement in enhancing both strength and ductility.
4. The hybrid mixture containing 5% PVC powder and 0.5% recycled polyester fiber achieved a 53.7% increase in unconfined compressive strength compared with the untreated clay. Although its peak strength was lower than that of the optimum individual mixtures, the hybrid system exhibited a balanced stress–strain response by combining improved strength with moderate deformation capacity. This response is interpreted as complementary functionality rather than a synergistic or super-additive strength effect.
5. SEM observations indicated that PVC powder contributed to void filling and particle rearrangement, whereas polyester fibers improved mechanical interlocking and crack-bridging behavior. However, because the highest-performing 15% PVC and 1.0% fiber mixtures were not examined by SEM, further microstructural investigation is recommended to directly establish the relationship between microstructural characteristics and maximum mechanical performance. Moreover, quantitative SEM image analysis was not performed; therefore, the relationship between microstructural descriptors and macroscopic mechanical parameters remains qualitative and should be investigated systematically in future studies.
6. The experimental results demonstrate that recycled PVC powder and polyester fibers can serve as effective and sustainable stabilization materials for high-plasticity clay. Beyond improving engineering performance, their use provides an environmentally responsible alternative for the beneficial utilization of polymer-based waste materials in geotechnical and transportation infrastructure applications. From a practical engineering perspective, the results indicate that additive selection should be based on the targeted performance requirement rather than strength alone. Within the investigated dosage range, 15% PVC may be considered where maximizing short-term compressive strength is the primary objective, although its reduced deformation capacity should be taken into account. In contrast, 1.0% recycled polyester fiber may be more suitable where a combination of strength and deformation capacity is required. The 5% PVC + 0.5% fiber mixture may provide an alternative when a balanced mechanical response is desired. These recommendations are limited to the investigated high-plasticity clay and laboratory conditions; therefore, field-scale validation, durability assessment, and site-specific testing are required before design-level implementation.

A further limitation of the present study is the use of a single 24 h conditioning period. Although sealed storage was adopted to facilitate moisture redistribution and maintain consistent conditions among specimens, this duration may not ensure complete internal moisture equilibration in a highly plastic clay (PI = 97%). Therefore, the reported UCS values should primarily be interpreted as short-term mechanical responses under the investigated conditioning condition. Longer conditioning periods may influence moisture redistribution and the resulting mechanical behavior even in systems modified predominantly through physical mechanisms. Future studies should therefore evaluate extended conditioning periods, including 7 and 28 days, to assess the time-dependent stability of the observed improvements.

Accordingly, future studies should systematically investigate a broader range of PVC–fiber combinations using factorial, response-surface, or other optimization approaches to identify formulations that provide an improved balance among compaction characteristics, strength, stiffness, and ductility.

## Figures and Tables

**Table 1:** Comparative summary of previous studies on polymer-based soil stabilization and their relevance to the present study.

Study	Additive and Form	Soil Type	Key Findings/Contribution	Limitation Relevant to the Present Study
Consoli et al. (2002) [21]	Plastic waste/discrete reinforcement	Sand	Plastic waste reinforcement modified the mechanical response of the soil and demonstrated the potential of waste polymers for geotechnical reinforcement.	Focused on sand and discrete plastic reinforcement rather than finely ground PVC powder or clay.
Kumar et al. (2007) [22]	Polyester fibers combined with fly ash and lime	Expansive soil	Polyester fibers contributed to improvements in the compaction and strength characteristics of stabilized expansive soil.	Fibers were incorporated with conventional stabilizers; particulate PVC–fiber interaction was not investigated.
Ural et al. (2020) [23]	Waste PVC	Soil	Demonstrated the potential applicability of waste PVC for soil improvement.	Did not systematically investigate the complementary behavior of finely ground PVC powder with recycled polyester fibers.
Khalid and Alshawmar (2024) [24]	Recycled PET/fibers and strips	Various soils (review)	Reported that recycled PET fibers and strips can improve several geotechnical properties, with performance depending on additive geometry, content, and soil conditions.	Focused primarily on PET fibers and strips rather than finely ground PVC powder and PVC–polyester hybrid stabilization.
Boukhatem et al. (2025) [25]	PVC additive	Clayey soil	Demonstrated that PVC incorporation can modify the geotechnical properties of clayey soil and that performance is dependent on PVC content.	Did not examine a hybrid system combining PVC powder with recycled polyester fibers.
El Majid et al. (2025) [26]	Recycled polypropylene fibers	Expansive soil	Fiber reinforcement improved the mechanical performance of expansive soil and supported the environmental benefits of recycled polymer utilization.	Investigated fiber reinforcement rather than a particulate polymer–fiber hybrid system.

**Table 2:** Mixture design adopted in the experimental program.

Mixture ID	Clay (%)	PVC Powder (%)	Polyester Fiber (%)
C	100	0	0
P5	95	5	0
P10	90	10	0
P15	85	15	0
F0.5	99.5	0	0.5
F0.75	99.25	0	0.75
F1.0	99	0	1
PF	94.5	5	0.5

Note: C = untreated clay; P5, P10, and P15 = clay amended with 5%, 10%, and 15% PVC powder, respectively; F0.5, F0.75, and F1.0 = clay reinforced with 0.5%, 0.75%, and 1.0% polyester fibers, respectively; PF = hybrid mixture containing 5% PVC powder and 0.5% polyester fiber. All percentages are expressed by dry weight of the soil.

**Table 3:** XRD-identified crystalline phases and main observations for untreated and treated clay specimens. (✓: indicates that the corresponding component is present.)

Sample	Illite	Quartz	Kaolinite	Calcite	Main XRD Observation
Untreated clay	✓	✓	✓	✓	Original mineralogical composition
Clay + 5% PVC	✓	✓	✓	✓	Main clay mineral phases preserved
Clay + 0.5% fiber	✓	✓	✓	✓	No change in identified principal phases
Clay + 5% PVC + 0.5% fiber	✓	✓	✓	✓	Original crystalline mineral assemblage retained

## Data Availability

The raw data supporting the conclusions of this article will be made available by the authors on request.
